# In this issue of *Current Oncology*

**Published:** 2010-02

**Authors:** M. McLean

This first issue of 2010 sees a gathering of momentum as *Current Oncology* transitions to hybrid-based publication. The “hybrid” format, a concept pioneered in recent years by the journal, allows for a significantly shorter delay to both online and hardcopy publication for authorship.

All manuscripts published as abstracts in print will be simultaneously published in full online at www.current-oncology.com in Portable Document Format (PDF), with subsequent indexing in PubMed, Excerpta Medica, and so on. We therefore encourage submissions to the journal to be accompanied by abstracts that fully reflect the scholarly content of the work. Some manuscripts that are deemed to be of a wider interest may appear in full in print on the recommendation of the reviewers and the editors, but the main intent remains to see the wait time to publication shorten substantially for all submissions—and indeed, this is already happening.

As I write this, approximately 40 additional manuscripts are in the publication “queue” at various stages of review and editing under the guidance of Dr. Martin Chasen. And notably, 14 original manuscripts appear in this issue, the largest number to date in nearly 17 years of publication of this journal.

Publication online allows for the addition of audio and video files—or of other media—to enhance published articles, and the journal welcomes those who might seek to pioneer the use of such tools. Although these enhancements might more readily be used by an ornithology journal, we welcome innovation nonetheless!

In the coming year, *Current Oncology* will continue to publish its very successful educational supplement series. Two more of these issues—on the subjects of colorectal cancer and management of chronic myelogenous leukemia—are already in the pipeline. I welcome correspondence with suggestions about subject matter for possible inclusion in this series, particularly from authors willing to take an active role in their planning or execution.

Lastly, my thanks go to the authors who have allowed us to change the presentation of their submissions as we continue to “hybridize.” Your patience and understanding has been greatly appreciated.

